# Effectiveness and Safety of Tuina Therapy Combined With Yijinjing Exercise for Neck Pain: Protocol for a Systematic Review and Meta-Analysis

**DOI:** 10.2196/77864

**Published:** 2025-09-17

**Authors:** Shichang Yang, Yuanming Zhong, Qipeng Yuan, Jinxu Wang, Guancheng Wang, Yan Long, Haoran Sun, Jie Si, Jinyu Li

**Affiliations:** 1 The First Affiliated Hospital of Guangxi University of Traditional Chinese Medicine Guangxi University of Traditional Chinese Medicine Nanning China

**Keywords:** massage, traditional Chinese exercise, cervical spondylosis, systematic review, meta-analysis

## Abstract

**Background:**

Neck pain with high incidence and recurrence rates significantly impairs patients’ quality of life and imposes a considerable economic burden. Traditional Chinese medicine therapies such as Yijinjing exercise and Tuina have shown promising efficacy in alleviating the local symptoms of neck pain. However, there is currently insufficient high-level evidence to robustly support these findings.

**Objective:**

This study aims to evaluate the efficacy and safety of combining Yijinjing exercise with Tuina for the treatment of neck pain.

**Methods:**

PubMed, Cochrane Library, Embase, Web of Science, China National Knowledge Infrastructure, Chinese Biomedical Database, VIP Chinese Science and Technology Periodicals Full-Text database, and Wanfang database will be systematically searched for all relevant randomized controlled trials (RCTs) from their inception to September 2025, without language or publication status restrictions. The Cochrane Risk of Bias 2 assessment tool will be used to evaluate the risk of bias in the included studies, and the GRADE (Grades of Recommendation, Assessment, Development, and Evaluation) system will be employed to grade the quality of evidence. Heterogeneity will be evaluated through *I*^2^ statistics and Cochran’s Q test: a fixed-effect model will be used when *I*^2^<50% and *P*≥.01. If *I*^2^≥50% or *P*<.01, subgroup analysis will be conducted. When heterogeneity still exists, sensitivity analysis or exploratory subgroup analysis will be performed. If it cannot be explained ultimately, the random-effects model will be adopted and the GRADE evidence level will be reduced.

**Results:**

As of June 2025, we have completed the preliminary screening of titles and abstracts for 573 studies. The full-text screening is expected to be completed by September 2025, and data analysis is planned to be completed by December 2025. About two-thirds of the studies were published after 2015. Geographically, the samples in the studies were highly concentrated in Asia. The results were comprehensively developed around the core outcomes. The primary outcome was presented by changes in the visual analog scale. The secondary outcomes were evaluated by the neck disability index, self-rating anxiety scale score, mean vertebral artery blood flow velocity, and Cobb angle.

**Conclusions:**

If the results of this study confirm the effectiveness of massage combined with Yijinjing, it can provide a direction for the nonpharmaceutical treatment of neck pain. However, some studies have risks of bias such as insufficient standardization of massage operations and difficulty in implementing blinding methods. The expected heterogeneity is significant due to differences in intervention plans and patients’ cultural backgrounds, and the original RCTs are few and regionally concentrated, with limited extrapolation of conclusions. In the future, it is necessary to optimize the plan and supplement data through high-quality multicenter research to enhance reliability.

**Trial Registration:**

PROSPERO CRD420251026508; https://www.crd.york.ac.uk/PROSPERO/view/CRD420251026508

**International Registered Report Identifier (IRRID):**

DERR1-10.2196/77864

## Introduction

Neck pain is the fourth leading cause of disability, with an annual prevalence of over 30% [1-3]. By 2050, the number of neck pain cases is projected to reach 269 million worldwide due to population growth and aging [4]. After the early acute neck pain ends, about half of the patients still have recurrent frequent pain [5]. The etiology and pathogenesis of neck pain are multifactorial, primarily associated with muscle strain, soft tissue injury, cervical facet joint disorders, and cervical disc degeneration [6,7]. The clinical symptoms and signs of neck pain are complex; therefore, its therapy often involves comprehensive intervention of multiple disciplines, but there is no complete unified treatment method [8,9]. Combination therapy is commonly used in the clinical treatment of neck pain, including medication, acupressure, cervical region exercises, psychological counseling, acupuncture, laser therapy, or traction [10]. Moreover, clinical treatment combined with functional exercise is absolutely necessary for neck pain [11,12].

Tuina, a form of manipulative therapy, is widely utilized in China for neck pain management due to its efficacy, cost-effectiveness, and noninvasiveness [13,14]. Tuina for the treatment of soft tissue injury and spinal degeneration has a wide range of effects such as reducing inflammation and swelling, separating adhesions, enhancing microcirculation, relieving spasm, alleviating pain, correcting small joint disorders, and improving the subhealth state [15,16]. Yijinjing is a traditional Chinese exercise that enhances the motor function and flexibility of the limbs through breathing and slow movements, reduces pain caused by joint pathology and stress [17,18], and helps in the later stages of recovery from several illnesses such as cardiovascular, psychiatric, and respiratory disorders [19,20]. For patients with neck pain, Yijinjing can regulate the muscle groups and improve skeletal muscle contraction and ligament strength, thus promoting blood circulation and achieving pain improvement. Following the completion of Tuina, instructing patients to perform Yijinjing exercise at home may enhance the therapeutic efficacy for neck pain and alleviate psychological issues such as anxiety and depression associated with prolonged pain [21,22]. Additionally, active participation in rehabilitation exercises can help patients build confidence in managing their condition, fostering a more positive attitude toward coping with neck pain [23,24].

At present, the combination of massage and Yijinjing exercise for the treatment of neck pain has been widely applied in clinical practice. Multiple clinical observations suggest that it has potential in relieving pain and improving cervical spine function [25]. However, the existing evidence system has obvious deficiencies. A search of the Cochrane Library, PubMed, and Chinese databases revealed that systematic reviews on neck pain mostly focused on single therapies and did not cover the abovementioned combination regimens [13,26]. To the best of our knowledge, to date, there is no systematic review or meta-analysis on the combination of massage and Yijinjing, and there is a lack of high-quality clinical research to support its safety and efficacy [27]. This evidence gap makes it difficult to fully verify its clinical value and limits its standardized application in clinical practice. To this end, this study intends to strictly screen the literature (only including randomized controlled trials [RCTs]), use the Cochrane Risk of Bias (RoB2) tool to evaluate the quality of the study, and apply the GRADE (Grades of Recommendation, Assessment, Development, and Evaluation) system to grade the strength of evidence, aiming to comprehensively and objectively integrate the clinical efficacy and safety of this combination therapy. We also aim to make up for the deficiencies of the existing studies [5,28] in the evaluation of combination regimens, methodological rigor, and evidence integration and to provide reliable evidence-based basis for clinical decision-making.

## Methods

### Study Registration

This systematic review protocol was developed following the guidelines of PRISMA-P (Preferred Reporting Items For Systematic Reviews and Meta-Analyses Protocols) (Multimedia Appendix 1). It has been registered in the PROSPERO database (registration CRD 420251026508). This protocol provides detailed descriptions focusing on research criteria, database selection, data collection, and bias assessment to ensure that the systematic review is objective, fair, transparent, and reproducible. The protocol will be updated on the PROSPERO database in the event of any methodological changes.

### Ethics and Dissemination

The required data will be obtained from published studies rather than from patients; therefore, ethical approval is not required for this study. The results of this systematic review will elucidate the efficacy and safety of Tuina combined with Yijinjing for the treatment of neck pain, which facilitates clinicians to make treatment decisions.

### Inclusion Criteria

The inclusion criteria will be determined using the PICOS (Population, Intervention, Comparison, Outcomes, and Study design) framework.

#### Population

The individual participants are aged between 20 and 70 years, with a visual analog scale (VAS) score over 3 points, a neck disability index (NDI) score over 10 points, and with no history of shoulder or neck surgery. The inclusion criteria were set at an age range of 20 to 70 years, as the cervical vertebrae of those younger than 20 years are still developing, and the causes of pain are different from those of middle-aged and older adults. People older than 70 years often have underlying diseases, which can easily interfere with the assessment. A VAS score greater than 3 indicates that pain significantly affects function and quality of life and can be used as a basis for evaluating treatment effects and clinical intervention. An NDI score greater than 10 indicates that the patient’s daily activities are restricted, which is in line with the core objective of the research to assess the impact of neck pain on life status. Having no history of shoulder and neck surgery can avoid the anatomical and physiological changes caused by surgery and the interference of complex pain mechanisms, thereby accurately determining the relationship between intervention measures and primary neck pain.

#### Intervention

Patients in the experimental group performed Tuina therapy combined with Yijinjing exercise at least once a week.

#### Comparison

Patients in the control group only received Tuina therapy.

#### Outcome

The primary outcome was the change in the VAS score after the intervention. Secondary outcomes encompassed the NDI score, self-rating anxiety scale score, average vertebral artery flow velocity, and Cobb angle. The Cobb angle, defined as the angle formed between the vertical lines of the lower endplates of C2 and C7 on the lateral cervical radiograph, serves as the standard for quantifying and monitoring scoliosis progression.

#### Study Design

The design of the study was an RCT of Tuina combined with Yijinjing for neck pain, regardless of sample size, publication status, or language.

### Exclusion Criteria

Non-RCTs, including reviews, case reports, observational studies, conference papers, and duplicate reports, are excluded. Patients with specific cervical spine conditions such as nerve root injury or spinal cord injury, pregnant or breastfeeding women, those who experienced major trauma or tumor-related major diseases, those unable to communicate verbally, those who develop adverse reactions to Tuina and Yijinjing, those who are unable to cooperate with the treatment, or those who refuse to self-exercise are excluded.

### Search Strategy

According to the inclusion criteria, PubMed, Cochrane Library, Embase, Web of Science, China National Knowledge Infrastructure, China Biomedicine database, VIP Chinese Science and Technology Periodicals Full-Text database, and Wanfang Data Knowledge Service Platform will be searched for RCTs published from database inception to September 2025. The search strategy combines P + I + S blocks by using medical subject headings (MeSH) and text words, with “OR” logic for broader scope and “AND” logic for precision. To obtain the latest evidence, the research team will reconduct the search at 1 month before submission and extend the timeline to include studies published before this date. PubMed search terms are provided in Table 1.

**Table 1:** PubMed search terms.

Search block	Search items
Participants	“Neck pain [MeSH^a^ Terms]” OR “Nonspecific neck pain [Title/Abstract]” OR “Neck pain [Title/Abstract]”
Intervention	“Yijinjing [MeSH Terms]” OR “Yijinjing [Title/Abstract]” OR “Chinese medicine exercise [Title/Abstract]” OR “Tuina [Title/Abstract]” OR “Massage [Title/Abstract]” OR “Chinese massage [Title/Abstract]”
Study design	“Randomized controlled trial [Title/Abstract]” OR “Controlled clinical trial [Title/Abstract]” OR “Clinical trial as topic [MeSH Terms]” OR “Randomized [Title/Abstract]” OR “Randomly [Title/Abstract]” OR “Trial [Title/Abstract]”

^a^MeSH: medical subject headings.

### Study Selection and Data Extraction

#### Study Selection

**Figure 1:**
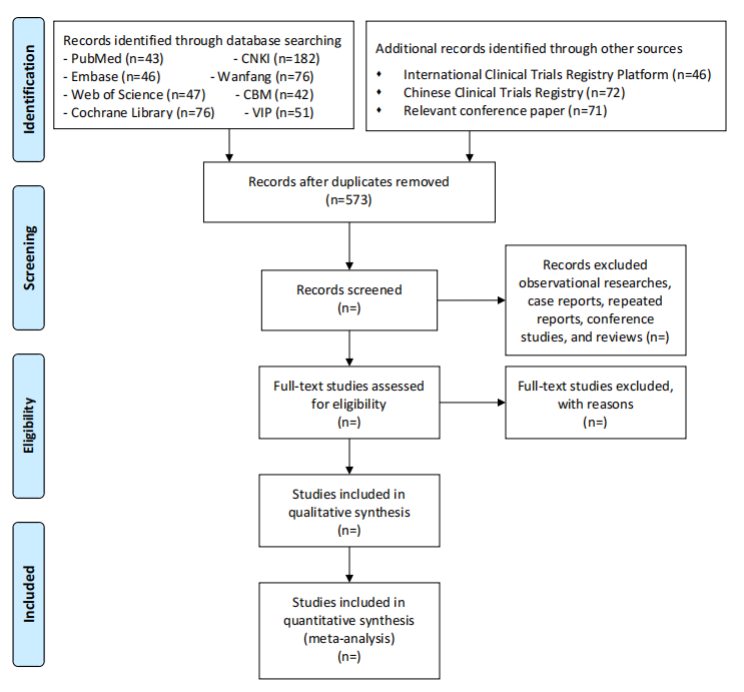
PRISMA (Preferred Reporting Items For Systematic Reviews and Meta-Analyses) flow diagram of this study. CBM: Chinese Biomedical Database; CNKI: China National Knowledge Infrastructure; VIP: VIP Chinese Science and Technology Periodicals Full-Text database.

Two researchers (JS and JL) will independently screen studies, removing duplicates and ineligible studies based on titles and abstracts. The screening of differences is resolved through the following process. First, JS and JL negotiate and reach a consensus by comparing the inclusion and exclusion criteria with the original text. If the negotiation fails, the case will be submitted to a senior supervisor (YZ) with over 10 years of experience in traditional medical research. The arbitration will be based on the consensus in the field and the research design. The basis for the arbitration needs to be recorded and archived. If there are still disputes, the research team will be organized to hold a special meeting, collectively discuss and determine the plan, and form meeting minutes for retention. The PRISMA flow diagram illustrates the selection process (Figure 1).

#### Data Extraction

During the data extraction stage, we will use a structured table system to collect key information that may cause methodological differences across studies. Massage techniques need to record item by item the type of technique (rolling method, pressing method, etc), application site (cervical pinch muscle, Fengchi point, etc), force level (light, moderate, heavy), operation frequency (times per day or times per week), and the duration of each session (minutes). The Yijinjing plan covers core movement combinations (such as Weituo Presenting the Pestle and Zhaixing Changing the Dou), practice time, frequency, and guidance form (professional guidance or self-study) and uniformly adopts classification by continuous or grade coding to achieve standardized recording. Data will be extracted using NoteExpress and Excel by 2 independent researchers (YL and HS) and stored in a spreadsheet. Extracted data include authors, publication year, patient demographics, study population, interventions, and outcomes. In case of any disagreement during the execution process, the 2 execution personnel will first negotiate together. If no agreement is reached through negotiation, it will be submitted to a senior supervisor (YZ) with over 10 years of experience in traditional medical research. The supervisor will arbitrate based on the consensus in the field or the requirements of the research design, clarify the arbitration basis, and file it. If there are still disputes after arbitration, the research team will be organized to hold a special meeting, determine the final plan through collective discussion, and form meeting minutes for retention.

### Risk of Bias Assessment

Two reviewers (SY and QY) will use Cochrane RoB2 to assess the risk of study bias and conduct a 3-level determination of low risk, partial risk, and high risk referring to the tool manual in 5 areas: randomization process, deviation from expected intervention, missing outcome data, outcome measurement, and selective reporting of results. The GRADE system was applied to grade the quality of evidence. Before the evaluation, 3 literature pretrials were conducted for calibration (target κ≥0.80). During the evaluation, factors such as the risk of bias, consistency, accuracy, and publication bias were independently and blindly evaluated. The quality of evidence was classified into 4 levels: high, medium, low, and very low. In case of disagreement, third-party arbitration was conducted.

In certain circumstances such as a large effect size, a clear dose-response relationship, or a scenario where all plausible biases would attenuate the effect estimates, the quality of evidence may be upgraded. For instance, if several included studies demonstrate a very large effect size or if an increase in the frequency or intensity of Yijinjing practice is associated with an increase in pain relief, these scenarios may support an upgrade in the quality of evidence. The quality of evidence is often downgraded due to factors such as research limitations, inconsistent results, circumstantial evidence, and publication bias. The quality of the evidence will also be downgraded if more than half of the studies have a high risk of bias in the Cochrane RoB2 assessment; if heterogeneity remains significant after subgroup analysis (*I*^2^≥50% and Cochran’s Q test *P*<.01) and cannot be explained by clinical factors; if there are large differences in study populations, interventions, and the target question; or if obvious publication bias (eg, asymmetric funnel plots or failure to report negative results) is present.

Disagreements are resolved through discussion or third-party adjudication (JW). Before the formal evaluation, some randomly selected studies will be pre-evaluated to unify the evaluation criteria. The data from the included studies will be used for subsequent meta-analysis or bias risk analysis. Further, the bias risk assessment results of all the included studies will be presented in the form of tables or charts.

### Statistical Analysis

Data analyses will be performed using Review Manager (RevMan) 5.4. For continuous outcome measures (such as VAS score and NDI score), the mean difference and 95% CI are calculated. Heterogeneity is evaluated using the *I*^2^ statistic and Cochran’s Q test. If *I*^2^<50% and Cochran’s Q test *P*≥.01, it indicates that heterogeneity can be ignored, and the fixed-effect model is used to combine the effect size. If *I*^2^≥50% or Cochran’s Q test *P*<.01, indicating substantial heterogeneity, stratified analysis will be conducted according to the preset subgroups. Subgroups include (1) characteristics of intervention implementation, such as the frequency of Yijinjing training (≥2 times/week vs <2 times/week) and massage treatment courses (<4 weeks vs ≥4 weeks); (2) population characteristics such as disease duration (<3 months vs ≥3 months), age (20-40 years vs 41-70 years), and NDI baseline score (10-20 points vs >20 points); and (3) study methodological characteristics such as blinding quality (double-blind/single-blind vs nonblind) and sample size (<100 cases vs ≥100 cases). The fixed-effect model was preferred within the subgroups. If in a subgroup *I*^2^<50% and Q≥0.1 but the clinical or methodological differences remain significant, a sensitivity analysis with item-by-item exclusion should be conducted first. After discovering new sources of heterogeneity, exploratory subgroups are established and labeled as post-hoc analyses. If heterogeneity still cannot be explained, a random-effects model will be adopted, and the evidence level will be downgraded according to GRADE.

### Multiple Test Correction

To control the risk of type I errors caused by multiple tests, in this study, the Bonferroni correction strategy is adopted for the intergroup difference analysis of 5 secondary outcome indicators (NDI score, self-rating anxiety scale score, average vertebral artery flow velocity, and Cobb angle). If the interaction effect test is included in the subgroup analysis, the same correction method must also be applied based on the actual number of tests.

Suppose significant heterogeneity still exists after subgroup analysis (*I*^2^≥50% and Cochran’s Q test *P*<.01), sensitivity analysis will be conducted. The stability of the results will be evaluated by eliminating low-quality studies (determined as high-risk bias based on the Cochrane RoB tool), excluding small sample studies (sample size <50 cases) or using the leave-one-out method to eliminate individual studies one by one, recalculating the combined effect size. If heterogeneity remains unresolved after sensitivity analysis, a metaregression will be conducted using the metareg command in Stata software to explore the contribution of the continuous variables (such as the average age of patients, disease duration, and intervention frequency) to heterogeneity. The significance of the covariates is determined by *P*<.05.

### Publication Bias

If fewer than 10 RCTs are included, publication bias will be assessed using funnel plots. If more than 10 RCTs are included, Egger regression test will be used for the quantitative analysis.

## Results

This systematic review was funded in August 2024. Starting from December 2024, specific databases were used for study retrieval: PubMed, Cochrane Library, Embase, Web of Science, China National Knowledge Infrastructure, Chinese Biomedical Database, VIP Chinese Science and Technology Periodicals Full-Text database, and Wanfang database. As of June 2025, after the initial search and deduplication, a total of 573 studies have entered the title and abstract screening stage. The full-text screening is expected to be completed by September 2025. Subsequently, rigorous data extraction and quality evaluation will be conducted. Data analysis is planned to be completed in December 2025. RevMan will be used in combination with the GRADE system and the RoB 2.0 tool to complete the quantitative synthesis of outcome measures and the grading of evidence quality.

A preliminary review of 573 search records revealed that previous research mainly focused on nonpharmaceutical interventions: approximately 45%-50% explored massage only, about 5%-8% used Yijinjing only, and less than 5 RCTs focused on the combination of massage and Yijinjing. Rest of the studies include acupuncture and exercise therapy. In terms of the time trend, approximately two-thirds of the studies were published after 2015. At the geographical level, about two-thirds of the studies were from Asia (especially China), <30% of the studies were from Europe and America, while there were relatively scarce number of studies from Africa and South America. The above distribution indicates that the original RCTs of this combined intervention are scarce and regionally concentrated, further supporting the necessity of this systematic review.

## Discussion

Neck pain is a globally prevalent chronic pain condition with high prevalence and recurrence rates that significantly impair patients’ quality of life. Currently, various therapeutic approaches are used to treat neck pain; yet, no standardized therapy has been established [29]. Common treatments include pharmacotherapy, physiotherapy, massage, acupuncture, and exercise [30]. In modern clinical medicine, nonsteroidal anti-inflammatory drugs combined with physical therapy are usually used to treat neck pain through analgesic and anti-inflammatory effects. However, long-term administration of nonsteroidal anti-inflammatory drugs may cause gastrointestinal discomfort and other adverse reactions. Additionally, this approach demonstrates definite therapeutic efficacy for acute neck pain conditions such as trauma or acute sprain. As for chronic neck pain and cases with complex symptoms, clinical practice often employs multimodal nonpharmacological therapies, including acupressure, psychological counseling, acupuncture, laser therapy, or traction [31]. Notably, traditional Chinese medicine therapies are widely used to treat neck pain because of their noninvasiveness, low toxicity, minimal side effects, and significant clinical efficacy [28,32].

Although there are several therapies available for neck pain, the treatment of neck pain still presents significant challenges. Due to the large individual differences of various treatment methods among patients with neck pain, the improvement of cervical pain symptoms in some patients is limited [33,34]. Tuina fully implements the traditional Chinese medicine concept of “blockage causes pain, while patency relieves pain” through the manipulation of soft tissue release and joint reduction. Its primary advantage resides in the capacity for immediate physical intervention, which directly releases local tissue adhesion and addresses mechanical disorders through manipulation, thereby rapidly alleviating pain symptoms. However, a few manipulation therapies in Tuina have inherent risks, for instance, cervical manipulation may worsen neck pain or cause damage to the vertebral arteries [35]. In addition, current treatment programs often overemphasize physician-led interventions and overlook the importance of patients’ self-exercise and timely feedback on treatment effects [36,37]. Therefore, effective therapies such as Tuina combined with self-exercise may provide better results for patients with neck pain. Different from modern self-exercise, traditional Chinese Yijinjing exercise with simplicity and wide applicability is suitable for patients of all ages with neck pain and will not be limited by environment and equipment [38,39]. As a representative of traditional Chinese dynamic exercises, the Yijinjing exercise has achieved continuous functional reshaping through a 3-in-1 intervention model of regulating the body, breath, and mind. This exercise method can effectively optimize the biomechanical state of the cervical vertebrae and play a regulatory role in neural regulatory function. Previous studies have proved that the combination of deep breathing and Yijinjing exercise can relieve neck muscle tension, promote blood circulation, and greatly improve patients’ negative emotions [40,41]. In addition, Yijinjing exercise combined with Tuina treatment can accelerate rehabilitation and improve the outcomes of patients with neck pain through a synergistic effect.

This study may encounter significant cross-trial heterogeneity, especially at the level of operational standardization of traditional therapies. The variations in massage techniques, force, body parts, and treatment courses can be as high as over 30%, and such operational differences may directly lead to systematic deviations in the evaluation of therapeutic effects. There are also significant differences in the guidance intensity, practice frequency, and compliance monitoring of Yijinjing, which may dilute or exaggerate the aggregated effect size. More crucially, the operator-dependent characteristics of traditional therapies are not limited to technical differences: the clinical experience of massage therapists and their ability to dynamically adjust to individual patient differences are both difficult to fully control through existing standardized means. This operational variability may lead to an increase in the amplitude of effect size fluctuations among studies (the expected *I*^2^ value may exceed 50%), and even with subgroup analysis or sensitivity analysis, it may still be impossible to completely eliminate its interference with the pooled results. Furthermore, this heterogeneity will directly limit the generalizability of the research conclusions. The intervention programs implemented by highly skilled massage therapists may have significantly higher effects than those in community medical institutions. Moreover, the differences in patients’ acceptance of traditional therapies from different cultural backgrounds (for instance, Asian patients may have a higher compliance with massage than those in Europe and America) further increase the difficulty of extrapolating the results. All these factors suggest that the meta-analysis results of this study should be interpreted with caution. The pooled effect size is more suitable as a theoretical reference value rather than a uniform clinical practice standard.

## Appendix

Multimedia Appendix 1 PRISMA-P checklist.

## Abbreviations

GRADE: Grades of Recommendation, Assessment, Development, and Evaluation
MeSH: medical subject headings
NDI: neck disability index
PICOS: Population, Intervention, Comparison, Outcomes, and Study design
PRISMA-P: Preferred Reporting Items For Systematic Reviews and Meta-Analyses Protocols
RCT: randomized controlled trial
RoB 2: Risk of Bias 2
VAS: visual analog scale

## Data Availability

The data that support the findings of this study are available from the corresponding author upon reasonable request.

## References

[1] Xia W, Liu J, Liu C, Xu S, Wang K, Zhu Z, Wang W, Wang H, Liu H, Zhou M (2024). Burden of neck pain in general population of China, 1990-2019: an analysis for the Global Burden of Disease Study 2019. J Glob Health.

[2] Cohen SP (2015). Epidemiology, diagnosis, and treatment of neck pain. Mayo Clin Proc.

[3] Cheng S, Cao J, Hou L, Li S, Sun W, Shan S, Zhao J, Yao L, Li X, He B, Song P (2024). Temporal trends and projections in the global burden of neck pain: findings from the Global Burden of Disease Study 2019. Pain.

[4] GBD 2021 Neck Pain Collaborators (2024). Global, regional, and national burden of neck pain, 1990-2020, and projections to 2050: a systematic analysis of the Global Burden of Disease Study 2021. Lancet Rheumatol.

[5] Reynolds B, McDevitt A, Kelly J, Mintken P, Clewley D (2025). Manual physical therapy for neck disorders: an umbrella review. J Man Manip Ther.

[6] Phrathep DD, Abdo Z, Tadros M, Lewandowski E, Evans J (2025). The role of osteopathic manipulative treatment for dystonia: a literature review. J Osteopath Med.

[7] Novais EJ, Narayanan R, Canseco JA, van de Wetering K, Kepler CK, Hilibrand AS, Vaccaro AR, Risbud MV (2024). A new perspective on intervertebral disc calcification-from bench to bedside. Bone Res.

[8] Kim R, Wiest C, Clark K, Cook C, Horn M (2018). Identifying risk factors for first-episode neck pain: a systematic review. Musculoskelet Sci Pract.

[9] Genebra CVDS, Maciel NM, Bento TPF, Simeão Sandra Fiorelli Almeida Penteado, Vitta AD (2017). Prevalence and factors associated with neck pain: a population-based study. Braz J Phys Ther.

[10] Mei F, Dong S, Li J, Xing D, Lin J (2023). Preference of musculoskeletal pain treatment in middle-aged and elderly Chinese people: a machine learning analysis of the China health and retirement longitudinal study. BMC Musculoskelet Disord.

[11] Sun X, Chai L, Huang Q, Zhou H, Liu H (2024). Effects of exercise combined with cervicothoracic spine self-mobilization on chronic non-specific neck pain. Sci Rep.

[12] Beltran-Alacreu H, López-de-Uralde-Villanueva Ibai, Fernández-Carnero Josué, La Touche R (2015). Manual therapy, therapeutic patient education, and therapeutic exercise, an effective multimodal treatment of nonspecific chronic neck pain: a randomized controlled trial. Am J Phys Med Rehabil.

[13] Yuan Q, Guo T, Liu L, Sun F, Zhang Y (2015). Traditional Chinese medicine for neck pain and low back pain: a systematic review and meta-analysis. PLoS One.

[14] Pach D, Piper M, Lotz F, Reinhold T, Dombrowski M, Chang Y, Liu B, Blödt Susanne, Rotter G, Icke K, Witt CM (2018). Effectiveness and cost-effectiveness of Tuina for chronic neck pain: a randomized controlled trial comparing Tuina with a no-intervention waiting list. J Altern Complement Med.

[15] Zhiwei WU, Qingguang Z, Lingjun K, Pengfei S, Xin Z, Guangxin G, Shuaipan Z, Tianxiang HE, Yanbin C, Min F (2024). Tuina alleviates neuropathic pain through regulate the activation of microglia and the secretion of inflammatory cytokine in spinal cord. J Tradit Chin Med.

[16] Chen L, Zhang Q, Huang Z, Da W, Liu S, Xue C, Ding C, Chen D, Fan T, Shi Q, Li X (2023). Efficacy of combining traditional Chinese manual therapy (Tuina) and specific therapeutic neck exercise in young adults with non-specific chronic neck pain: study protocol for a randomized controlled trial. J Pain Res.

[17] Liu X, Pan F, Wang Q, Wang S, Zhang J (2024). Traditional Chinese Rehabilitation Exercise (TCRE) for myofascial pain: current evidence and further challenges. J Pain Res.

[18] Guo G, Wang Y, Xu X, Lu K, Zhu X, Gu Y, Yang G, Yao F, Fang M (2024). Effectiveness of Yijinjing exercise in the treatment of early-stage knee osteoarthritis: a randomized controlled trial protocol. BMJ Open.

[19] Shi F, Yu J, Wang H, Wu C (2024). The impact of various mind-body exercises on cardiorespiratory function and quality of life in heart failure patients: a network meta-analysis. Curr Probl Cardiol.

[20] Li L, Liang J, Fan T (2025). Effect of five traditional Chinese medicine exercises on insomnia: a systematic review and network meta-analysis. J Psychiatr Res.

[21] Zhang X, Ye F, Yin Z, Li Y, Bao Q, Xia M, Chen Z, Zhong W, Wu K, Yao J, Liang F (2024). Research status and trends of physical activity on depression or anxiety: a bibliometric analysis. Front Neurosci.

[22] Chang T, Ma X, Gong X, Xia C, Jiang Q, Zhang R (2024). Effect of traditional Chinese Yijinjing exercise on hand dysfunction in rheumatoid arthritis patients: a randomized controlled trial. Front Med (Lausanne).

[23] Dickson C, de Zoete RMJ, Berryman C, Weinstein P, Chen KK, Rothmore P (2024). Patient-related barriers and enablers to the implementation of high-value physiotherapy for chronic pain: a systematic review. Pain Med.

[24] Deegan O, Fullen BM, Segurado R, Doody C (2024). The effectiveness of a combined exercise and psychological treatment programme on measures of nervous system sensitisation in adults with chronic musculoskeletal pain - a systematic review and meta-analysis. BMC Musculoskelet Disord.

[25] Cheng Z, Zhang S, Gu Y, Chen Z, Xie F, Guan C, Fang M, Yao F (2022). Effectiveness of Tuina therapy combined with Yijinjing exercise in the treatment of nonspecific chronic neck pain: a randomized clinical trial. JAMA Netw Open.

[26] Kong L, Ren J, Fang S, He T, Zhou X, Fang M (2022). Traditional Chinese exercises on pain and disability in middle-aged and elderly patients with neck pain: a systematic review and meta-analysis of randomized controlled trials. Front Aging Neurosci.

[27] de Oliveira-Souza AIS, Barbosa-Silva J, Gross DP, da Costa BR, Ballenberger N, Pereira TV, Dennett L, Armijo-Olivo S (2025). Comparative effectiveness of manual therapy, pharmacological treatment, exercise therapy, and education for neck pain (COMPETE study): protocol of a systematic review with network meta-analysis. Syst Rev.

[28] Fu L, Li J, Wu W (2009). Randomized controlled trials of acupuncture for neck pain: systematic review and meta-analysis. J Altern Complement Med.

[29] Cohen SP, Hooten WM (2017). Advances in the diagnosis and management of neck pain. BMJ.

[30] Fandim JV, Nitzsche R, Michaleff ZA, Pena Costa LO, Saragiotto B (2021). The contemporary management of neck pain in adults. Pain Manag.

[31] Dabbs V, Lauretti WJ (1995). A risk assessment of cervical manipulation vs NSAIDs for the treatment of neck pain. J Manipulative Physiol Ther.

[32] Wu Z, Kong L, Zhu Q, Song P, Fang M, Sun W, Zhang H, Cheng Y, Xu S, Guo G, Zhou X, Lv Z (2019). Efficacy of tuina in patients with chronic neck pain: study protocol for a randomized controlled trial. Trials.

[33] Pillastrini P, Castellini G, Chiarotto A, Fasciani F, Marzioni F, Vanti C, Bertozzi L, Gianola S (2019). Comparative effectiveness of conservative and pharmacological interventions for chronic non-specific neck pain: protocol of a systematic review and network meta-analysis. Medicine (Baltimore).

[34] Parikh P, Santaguida P, Macdermid J, Gross A, Eshtiaghi A (2019). Comparison of CPG's for the diagnosis, prognosis and management of non-specific neck pain: a systematic review. BMC Musculoskelet Disord.

[35] Smith WS, Johnston SC, Skalabrin EJ, Weaver M, Azari P, Albers GW, Gress DR (2003). Spinal manipulative therapy is an independent risk factor for vertebral artery dissection. Neurology.

[36] Wang R, Zhou C, Wu Y, Sun M, Yang L, Ye X, Zhang M (2022). Patient empowerment and self-management behaviour of chronic disease patients: a moderated mediation model of self-efficacy and health locus of control. J Adv Nurs.

[37] Wakaizumi K, Shinohara Y, Kawate M, Matsudaira K, Oka H, Yamada K, Jabakhanji R, Baliki MN (2024). Exercise effect on pain is associated with negative and positive affective components: a large-scale internet-based cross-sectional study in Japan. Sci Rep.

[38] Yang F, Zhang J (2022). Traditional Chinese sports under China's health strategy. J Environ Public Health.

[39] Neumann RJ, Ahrens KF, Kollmann B, Goldbach N, Chmitorz A, Weichert D, Fiebach CJ, Wessa M, Kalisch R, Lieb K, Tüscher O, Plichta MM, Reif A, Matura S (2022). The impact of physical fitness on resilience to modern life stress and the mediating role of general self-efficacy. Eur Arch Psychiatry Clin Neurosci.

[40] Zhang S, Guo G, Li X, Yao F, Wu Z, Zhu Q, Fang M (2021). The effectiveness of traditional Chinese Yijinjing Qigong exercise for the patients with knee osteoarthritis on the pain, dysfunction, and mood disorder: a pilot randomized controlled trial. Front Med (Lausanne).

[41] Cheng Z, Chen Z, Xie F, Guan C, Gu Y, Wang R, You Y, Yao F (2021). Efficacy of Yijinjing combined with Tuina for patients with non-specific chronic neck pain: study protocol for a randomized controlled trial. Trials.

